# The practical utility of psychometric scales for the assessment of the impact of posttraumatic scars on mental health

**DOI:** 10.3389/fpubh.2023.1103714

**Published:** 2023-04-05

**Authors:** Gabriel Mihai Mekereș, Camelia Liana Buhaș, Cristina Tudoran, Andrei Nicolae Csep, Mariana Tudoran, Felicia Manole, Claudiu Sorin Iova, Nicolae Ovidiu Pop, Ioan Bogdan Voiţă, Daniela Domocoș, Florica Voiţă-Mekereş

**Affiliations:** ^1^Faculty of Medicine and Pharmacy, University of Oradea, Oradea, Romania; ^2^Department VII, Internal Medicine II, University of Medicine and Pharmacy “Victor Babes” Timisoara, Timisoara, Romania; ^3^Center of Molecular Research in Nephrology and Vascular Disease, Faculty of Medicine, University of Medicine and Pharmacy “Victor Babes” Timisoara, Timisoara, Romania; ^4^County Emergency Hospital “Pius Brinzeu”, Timisoara, Romania; ^5^Regional Institute of Gastroenterology and Hepatology “Prof. Octavian Fodor”, Cluj-Napoca, Romania

**Keywords:** posttraumatic scar, psychometric scales, practical utility, evaluation algorithm, mental health

## Abstract

**Background:**

Scars are a natural consequence of the healing process, but with an impact on the psychological and social level for the individual, which can even lead to withdrawal and social stigmatization. We aimed to analyze the psychosocial impact determined by post-traumatic scars, using psychometric scales, to assess the effectiveness of the Mekereș’ Psychosocial Internalization Scale (MPIS), and to identify relevant predictors of traumatic and surgical scar internalization.

**Methods:**

Our cohort included 293 participants, 149 women and 144 men, aged 18–64 years who were screened for scar characteristics and completed a set of psychological scales. We compared the results obtained in two subgroups: 153 subjects with posttraumatic scars and 140 with surgical scars.

**Results:**

Relevant predictors for posttraumatic scar internalization (*R*^2^ = 0.721) are adaptation time, age of the occurrence and subjective appraisal, while for the depression, and hopelessness relevant predictors are the subjective appraisal of the scars and the posttraumatic quality of life.

**Conclusion:**

The psychological and social reporting of the aftermath of trauma, that has been followed by scar-ring, is an indicator of how a person will react and could indicate the susceptibility to psycho-pathology.

## 1. Introduction

People who present congenital or post-traumatically scars often face the challenges of social perception and their own psychological responses to their altered appearance. There is no simple linear relationship between the degree of disfigurement and the degree of suffering experienced by patients.

Among the factors that influence an individual’s ability to cope with the post-traumatic consequences, there are: the social significance of the aesthetic damage, his experiences, perceived social and family support at the time of the traumatic event and the stage of personality development.

A current issue are scar assessment tools that should be accurate and reliable, but there is a lack of consensus regarding the most appropriate and applicable assessment methods. Refinement of scar assessment methods will facilitate accurate analysis of treatment outcomes and enhance the ability to study scars (1).

Scales that evaluate scars were designed to quantify their appearance in response to treatment, but these scales are observer dependent, most taking into account factors such as scar height or thickness, flexibility, surface, texture, pigmentation and vascularity.

Surprisingly, little is known about how scars affect patients’ lives, although expert clinical impressions suggest that their impact is related to both their physical and psychosocial effects. Facial scars cause high levels of anxiety and low self-confidence (2).

Internalization is a peculiar adaptive process that appear during interhuman relationships, being the result of discipline and based on the accurate perception of messages determining their acceptance or rejection (3). In our study, scar internalization was conceptualized as the process by which people progressively accept the current situation and integrate it into their personality, so that their behavior becomes internally controlled or self-regulated, rather than externally controlled (3).

The extremely limited number of studies in this research niche on the internalization of posttraumatic scars often involved people of different ages and conceptualized internalization as compliance with the demands of people close or significant to them. Although most common definitions of internalization involve a process of transferring values from an external source of control or motivation to an internal source, few theories of internalization identify the sequences or levels of this process.

The concept of internalization brings together cognitive, motivational and socio-cognitive processes through which an external social value or norm becomes an internal requirement for the person, just as in the case of a traumatic event, if the child had good socio-familial support, the scars will be accepted easier as a component part of the body (4).

The internalization establishes standards for moral values, exigence and prosocial behavior, such as the pleasure of helping and giving to another person, opposed to aggressive behaviors. Psycho-social interventions are learned in the family, they are part of the cultural heritage of the community, group and family, being values that are formed and developed since childhood (5).

Aesthetic prejudice is a medico-legal term that refers to the impairment of the native physical appearance in general, not just the physiognomy of the face. The criteria for an injury to qualify as aesthetic damage are the following: obvious disharmony, irreversibility, native appearance changed for the worse.

Scars can constitute distinctive signs that can give useful information for identifying people because they remain strongly fixed in the memory of relatives or those close to them; the way of production (mechanical, physical, chemical, therapeutic agents, etc.), the time elapsed since the occurrence of the event, and their severity can determine psycho-emotional consequences (anxiety, depression), medico-legal (aesthetic prejudice), legal (criminal) and socio-economic (divorce, job loss) (6).

Based on these considerations, we thought to study and analyze the psychosocial effect of scars using psychometric scales and apply them to people with post-traumatic versus post-surgical scars.

The aim of this study is to highlight the practical utility of psychometric scales and thus to create an algorithm for evaluating scars in order to obtain the maximum of their early corrections, both physical and psychosocial corrections and to decrease internalization.

## 2. Manuscript formatting

### 2.1. Materials and methods

#### 2.1.1. Participants

The study included a total number of 293 participants, aged between 18 and 64 (*m* = 38.75; SD = 13.04), of which 149 were women (50.9%) and 144 were men (49.1%). The participants were divided into two relatively homogeneous groups, namely experimental with post-traumatic scars (*N* = 153; 52.2%) and control with surgical scars (*N* = 140; 47.8%).

The experimental group included a number of 153 participants aged between 18 and 64 years. The control group included a number of 140 participants aged between 18 and 64 years.

Inclusion criteria:

- participants who presented a post-traumatic or post-surgical scar located anywhere on the body;
- patients who did not present pre-existing dermatological pathology;
- age between 18 and 64 years at the time of examination;
- informed consent of the participants.

Exclusion criteria:

- participants who refused to participate in the study;
- age under 18 or over 64;
- uncontrolled mental illness;
- participants who did not know how to read the questionnaires and the informed consent.

#### 2.1.2. Instruments

Mekeres’ Psychosocial Internalization Scale (MPIS) takes into account the awareness of the presence of the scar, the sex of the victim, the morphological characteristics of the scar, the negative influence of social interaction and the impact on the professional development of the patient, etc. (3, 6).

The Patient and Observer Scar Assessment Scale (POSAS) scores scar characteristics such as vascularity, pigmentation, firmness, pliability, affected area and scar height, is a standardized and validated tool in the clinical, objective assessment of scars, also taking into account the patient’s symptoms related to the scar such as pain and itching, which were not considered in previous scales. In the study we used the PS subscale from POSAS (7).

The Hopelessness Depression Symptom Questionnaire (HDSQ) is an instrument that allows the examination of hopelessness depression symptoms individually or in groups. The instrument has eight subscales, each of which includes four items and each one measures a different symptom of hopelessness depression (8).

The Multidimensional Scale of Perceived Social Support (MSPSS) consists of 12 items targeting three factors: family, friends and significant others. Each item is structured according to these factors. The scales are anchored so that the highest scores reflect the highest perceived social support (9).

The EQ-5D is a standardized measure of health-related quality of life that can be used in clinical and economic evaluation and in population health surveys (10, 11). The descriptive system comprises five dimensions: mobility, self-care, usual activities, pain/discomfort and anxiety/depression.

#### 2.1.3. Working procedure

The study was carried out in County Emergency Clinical Hospital (CECH), Oradea with the approval of the Ethics Commission and in the individual medical office of Dr. Voiță Gheorghe Florin, Oradea with the agreement of the management of the office.

The patients were examined at the time of their presentation at the headquarters of Forensic Medicine Service of County Bihor, for the evaluation of some wounds on the body in order to obtain the medico-legal certificate or the examination of post-traumatic scars in order to be able to carry out the medico-legal expertise related to the previous traumatic event. Some of the participants in the study were evaluated in the CECH, Oradea, but also in the dental office of Dr. Voita Gheorghe Florin,” Oradea where the patients presented scars especially in the face region. All study participants read and signed the informed consent and received additional data if needed.

#### 2.1.4. Data analysis

In the first part of the research, we capture the initial statistical indicators of the variables recorded in the case of the experimental group with post-traumatic scar and in the case of the control group with post-surgical scar. We are interested in estimating the frequencies of the classifier variables that will be experimentally manipulated.

We analyze relevant data for demographic variables such as: age, gender, background, education level, profession, marital status and ethnicity.

From a clinical point of view, we analyze relevant data: type of scar (post-traumatic, surgical, congenital), mode of production (fall, aggression, accidents, etc.), age of production, number of scars (single or multiple), shape of scars (linear or non-linear), size, area, orbicular impairment, mimicry impairment and body symmetry impairment.

We analyze socio-occupational variables that could play an important role in understanding the impact of post-traumatic and surgical scars such as: time elapsed since scarring, adaptation time, social withdrawal, family relationships, relationships with work colleagues, change of workplace and the quality of being insured (health).

Greater attention is paid to the MPIS in which case we will use Exploratory Factor Analysis (as primary analysis) and Confirmatory Factor Analysis (through the AMOS statistical program as part of the SPSS 22 package) which will establish the validity of the tool (recently appeared and still insufficiently supported experimentally).

In the third part of the research we analyze the association (correlations) between psychosocial internalization of scars, depression of hopelessness, appreciation of scars and quality of life (mobility, personal care, usual activities, pain/discomfort and anxiety/depression). On the other hand, we analyze the association between the perception of social support (family, friends and significant others), the psychosocial internalization of scars, the patient’s appreciation of scars and components of quality of life (usual activities, pain/discomfort and anxiety/depression).

In the fourth part of the research, we identify the predictors that effectively support the estimation of the evolution of the dependent variable in the case of people with post-traumatic and surgical scars. In the first part, the dependent variable is the psychosocial internalization of the scars. Predictors are: scar adaptation time, age of scarring, hopelessness depression, POSAS patient.

In the second part the dependent variable is hopelessness depression. Predictors are: POSAS patient, personal care, usual activities, pain/discomfort. Considering the association between the variables presented during the research as well as the number of research participants we use the predictive regression equations to capture the effective predictors.

**Table 1:** Socio-demographical data of patients included in the study.

Characteristics		Experimental group (*n* = 153)	Control group (*n* = 140)	*t*/*χ*^2^	*p*
Age		38.15 ± 13.02	39.4 ± 13.07	0.819	0.413
Gender	Women	79 (51.6%)	70 (50%)	0.038	0.845
	Men	74 (48.4%)	70 (50%)	0.037	0.848
Marital status	Unmarried	44 (28.8%)	33 (23.6%)	0.258	0.611
	Married	80 (52.3%)	93 (66.4%)	3.538	0.060
	Divorced	17 (11.1%)	7 (5%)	0.209	0.647
	Widower	9 (5.9%)	6 (4.3%)	0.017	0.895
	Stable relationship	3 (2%)	1 (0.7%)	0.006	0.939
Geographical Distribution	Rural	74 (48.4%)	73 (52.1%)	0.200	0.654
	Urban	79 (51.6%)	67 (47.9%)	0.197	0.657
Education	Gymnasium	12 (7.8%)	10 (7.1%)	0.004	0.951
	High school	20 (13.1%)	24 (17.1%)	0.132	0.716
	College	71 (46.4%)	61 (43.6%)	0.103	0.748
	License	39 (25.5%)	32 (22.9%)	0.064	0.800
	Master or Doctorate	11 (7.2%)	13 (9.3%)	0.033	0.856
Ethnicity	Romanian	93 (60.8%)	91 (65%)	0.346	0.556
	Hungarian	35 (22.9%)	26 (18.6%)	0.163	0.686
	Roma	18 (11.8%)	14 (10%)	0.025	0.873
	Others	7 (4.6%)	9 (6.4%)	0.023	0.880
Health insurance	Health insurance	123 (82.4%)	109 (77.9%)	0.736	0.390
	Uninsured	27 (17.6%)	31 (22.1%)	0.111	0.739
Occupation	Workers	22 (14.4%)	28 (20%)	0.262	0.608
	Physicians	17 (11.1%)	10 (7.1%)	0.112	0.738
	Student	13 (8.5%)	10 (7.1%)	0.015	0.903
	Nurses	7 (4.6%)	6 (4.3%)	0.001	0.980
	Sellers	7 (4.6%)	5 (3.6%)	0.007	0.934
	Engineers	8 (5.2%)	8 (5.7%)	0.002	0.966
	Other	79 (52.6%)	8 (5.7%)	6.345	0.011

t, *t*-test; χ^2^, chi-squared test; *p*, statistical significance.

## 3. Results

### 3.1. Statistical indicators of post-traumatic and surgical scars

Analysis of recorded clinical and statistical data indicates 162 (55.3%) participants with posttraumatic scar and 131 (44.7%) participants with surgical scar. Multiple scars were reported by 121 (41.3%) participants and single scar by 172 (58.7%), on the other hand, frequency analysis of the recorded data indicates 129 (44%) participants with a linear scar and 164 (56%) with a non-linear scars and their characteristics are presented in Table 1. The recorded variability of the age at which the scars occurred (Table 2) is between 1 and 61 years (*m* = 25.66; SD = 14.38), and their sizes vary between 1 and 110 cm (14.37; SD = 14.04) with an area between 1 and 70 cm2 (*m* = 9.33; SD = 12.03).

**Table 2:** Frequency of ages by group type.

Variable	Category	Experimental group	Control group	*χ* ^2^	*p*
Age	1. Below 25 years old	25 (16.3%)	19 (13.6%)	0.000	1.000
	2. 26–35 years old	44 (28.8%)	42 (30%)	0.015	0.903
	3. 36–45 years old	49 (32%)	37 (26.4%)	0.314	0.575
	4. 46 years old and above	35 (22.9%)	42 (30%)	0.485	0.486

*χ*^2^, chi-squared test; *p*, statistical significance.

In Table 3 we present the recorded frequencies of the participants in the two groups regarding the way in which facial expressions and body symmetry are affected. We observe a more pronounced impairment of mimicry in the case of participants from the posttraumatic group (experimental; 73; 45.1%) compared to the control group (28; 21.4%). In addition, body symmetry was affected in the posttraumatic group (64; 39.5%) to a greater extent compared to the frequencies observed in the surgical group (25; 19.1%).

**Table 3:** Reported frequency of scarring in the two groups by affected area.

Group		Post-traumatic	Post-surgical	*χ* ^2^	*p*
Mimic affection	Yes	73 (45.1%)	64 (39.5%)	0.434	0.509
	No	89 (54.9%)	98 (60.5%)	0.597	0.439
Affecting body symmetry	Yes	28 (21.4%)	25 (19.1%)	0.042	0.837
	No	103 (78.6%)	106 (80.9%)	0.170	0.679

*χ*^2^, chi-squared test; *p*, statistical significance.

The scar location variable indicates the location on the body in 71 (43.8%) participants and on the face in 91 (56.2%) of the participants included in the experimental (posttraumatic) group, and in the control (surgical) group we observe the location of the scars on the body in 87 (66.4%) participants and in 44 (33.6%) participants the scars are located on the face.

The orbicular damage recorded in the case of the posttraumatic experimental group (N = 162) indicates 43 (26.5%) participants with scars present at the level of the eyes, 36 (22.2%) patients with scars at the level of the mouth and 83 (51.2%) without an orbicularis oculi lesion. Frequencies of participants with surgical scar and included in the control group indicate 23 (17.6%) participants with orbicular scar, 37 (28.2%) participants with surgical scar at the level of the mouth and 71 (54.2%) participants without a facial lesion.

The reported age of scarring is between 1 and 61 years (*m* = 25.66; AS = 14.38), however we also consider relevant the time elapsed since the scarring as well as the time needed to adapt to post-traumatic or surgical scars. Thus, the participants of the experimental group report between 1 and 40 years (*m* = 11.35; SD = 10.07) since the occurrence of the trauma, while the participants included in the control (surgical) group report between 1 and 53 years (*m* = 14, 52; SD = 12.73) from surgery. Comparison of the two groups indicates (*t*-test for independent samples) a statistically significant difference in time [*t*(291) = −2.337; *p* < 0.01] in that participants with surgical scar had a longer time span since the scars occurrence compared to the experimental group in which participants with posttraumatic scars were included (Table 4).

**Table 4:** Comparisons between the experimental group (*N* = 162) and the control group (*N* = 131) regarding adaptation time and time since scarring.

Group	Scar	*m*	SD	*t*	Df	*P*
Adaptation time	Post-traumatic	2.68	2.91	0.745	291	0.45
	Post-surgical	2.40	3.52			
Time since production	Post-traumatic	11.35	10.07	−2.377	291	0.01
	Post-surgical	14.52	12.73			

m, mean; SD, Standard Deviation; t, *t*-test; Df, Degrees of freedom; **p < 0.01 – statistical significance.

Participants were asked to estimate the time of adaptation to the scars. The values recorded in the case of the experimental group and the control group (Table 5) indicate values between 0 and 18 years (*m* = 2.68; SD = 2.91) in the case of the experimental group and in the case of the control group we record values between 0 and 20 years (*m* = 2.40; SD = 3.52) and the t-test does not indicate a significant difference in means [*t*(291) = 0.745; *p* = 0.45].

**Table 5:** Comparisons between the experimental group (*N* = 162) and the control group (*N* = 131) according to gender regarding the scales included in the study.

	Group	Gender	*N*	*M*	SD	*t*	Df	*p*
Experimental	MPIS	M	78	42.08	11.38	−4.172	160	0.001^***^
		F	84	49.78	12.04			
	HDSQ	M	78	15.2	18.33	−3.447	160	0.001^***^
		F	84	26.58	23.19			
	PS-Patient	M	77	29.23	18.50	−1.744	160	0.08
		F	84	34.48	19.62			
Control	MPIS	M	66	39.66	11.88	−0.439	129	0.66
		F	65	40.53	10.82			
	HDSQ	M	66	9.28	15.55	−0.369	129	0.71
		F	65	10.29	15.59			
	PS-Patient	M	66	20.1	11.61	−1.254	129	0.21
		F	65	23.07	15.27			

N, number; M, Mean; SD, Standard Deviation; *t*, *t*-test; df, Degrees of freedom; MPIS, Mekeres Psychosocial Internalization Scale; HDSQ, Hopelessness Depression Symptom Questionnaire; PS, Patient scale – Patient and Observer Scar Assessment Scale; **p < 0.01; ***p < 0.001.

The MPIS of scars was proposed as a tool for evaluating aesthetic damage and service, it indicates in the case of the post-traumatic group [*t*(160) = −4.172; *p* < 0.001] higher averages in women compared to men but not in the case of the control group where the means are close [*t*(129) = −0.439; *p* = 0.66].

The Hopelessness Depression Questionnaire (HDSQ) used as a depression reporter provides additional information regarding gender differences in the post-traumatic scar group in the sense that women compared to men show more intense post-traumatic depression symptoms [*t*(160) = −3.447; *p* < 0.001]. However, we do not record statistically significant differences in the control group [*t*(129) = −0.369; *p* = 0.77] where the post-surgical scars are expected by the participants and the psychological mechanisms, respectively the coping mechanisms work in a much more adaptive manner (Table 4).

The assessment of scars by the participants (POSAS) indicates a tendency to perceive with greater accuracy the scars in terms of color, regularity, etc., in women from the post-traumatic group compared to men [*t*(160) = −1.744; *p* < 0.08]. Evaluation of scars in the control group does not indicate relevant gender differences [*t*(129) = −1.254; *p* = 0.21].

The obtained results support the fact that in the case of our group the composition of the MPIS subscales is suitable, the items show validity in the case of the native population with scars, both in the case of adolescents and in the case of adults.

### 3.2. The relationship between perception of social support and evaluation of the impact of scars

Social support is considered an important resource that protects the person against physical or psychological threats. Progress is represented by the number of researches that focus on the need to distinguish between different levels of analysis in the field of social support (1).

Social support aims at interpersonal relationships characterized by the feeling of acceptance, esteem and appreciation, of belonging to a network of communication and mutual obligations, of emotional and concrete support in times of crisis (8, 12, 13). The degree of integration into a social network or structural support has been found to have a direct effect on “well-being,” reducing negative outcomes in both low and high stress circumstances (14).

Starting from the assumptions previously presented and demonstrated in psychological studies, we aimed to identify how the evaluation of post-traumatic and surgical scars is associated with the perception of social support.

In Table 6 we observe a distancing (dissociation) of the social support perceived by the participants from the internalization of scars (*r* = −0.22; *p* < 0.01). Participants’ distorted evaluation of scars (*r* = −0.19; *p* < 0.05) indicates a decrease in the perception of social support. In the case of the perception of support from family, friends or significant others, the presented results indicate a similar orientation of the results.

**Table 6:** Correlations between perceived social support and scales measuring the impact of scars in the case of the experimental group (*N* = 162).

Variable	Family support	Friends support	Support from significant others	Global social support
MPIS	−0.16^*^	−0.18^*^	−0.28^**^	−0.22^**^
POSAS P	−0.12	−0.17^*^	−0.25^**^	−0.19^*^
Usual activities	−0.20^**^	−0.20^**^	−0.23^**^	−0.22^**^
Pain/discomfort	−0.13	−0.17^*^	−0.24^**^	−0.19^*^
Anx./depr.	−0.17^*^	−0.20^**^	−0.24^**^	−0.22^**^

MPIS, Mekeres Psychosocial Internalization Scale; POSAS P, Patient scale – Patient and Observer Scar Assessment Scale; Usual activities – EuroQoL-5_3; Pain/discomfort – EuroQoL-5_4; Anxiety/depression – EuroQoL-5_5; *p < 0.05; **p < 0.01.

The coefficients presented in the case of people with surgical and post-traumatic scars are related to the concept of loneliness, thus differentiating two dimensions, namely: emotional and social loneliness. Social loneliness is associated with the absence of employment in the social network and predominantly with feelings of marginalization.

The availability of social support depends on the characteristics of people with scars as well as communication skills (15). Therefore, personal and social characteristics that make communication difficult are probably associated with psychopathological effects such as ambivalence in emotional expression, repressive defensiveness and fear of intimacy.

### 3.3. Predictions of prevalence by scar internalization in participants with posttraumatic and postsurgical scars

In the last part of the research, we aim to identify the predictors that estimate the evolution of scar internalization and, more precisely, which of the predictors are clinically / medically effective. The preliminary analyzes confirm compliance with the conditions regarding homogeneity and multicollinearity.

We postulate that time of adaptation to scars, age of scarring, hopelessness depression, patient assessment of scars (patient POSAS) are important predictors of scar estimation and internalization (MPIS) at least in posttraumatic research participants.

Table 7 summarizes the statistical differences recorded after the statistical processing of the results according to the presence or absence of internalization [*F*(160) = 79.987; *p* < 0.001] in the experimental group and the control group [*F*(130) = 11.986; *p* < 0.001].

**Table 7:** Coefficients of the factors included in the predictive multilinear regression equation in patients with scars (posttraumatic and surgical) according to psychosocial internalization (MPIS).

Model summary						
Group	*R*	*R* ^2^	Adjusted *R*^2^		*F*	
Experimental	0.849^a^	0.721	0.712		*F*_(160)_ = 79.987; *p* < 0.001	
Control	0.569^c^	0.324	0.297		*F*_(130)_ = 11.986; *p* < 0.001	
Group	Model	Unstandardized Coefficients		Standardized Coefficients	*t*	*p*
		*B*	Std. Error	Beta		
Experimental	(Constant)	25.53	1.49		17.073	0.001
	The depression of hopelessness	0.11	0.03	0.19	3.674	0.001
	Adaptation time	0.57	0.25	0.09	2.217	0.02
	Production age	−0.11	0.04	−0.13	−2.878	0.005
	POSAS P	0.18	0.03	0.28	5.067	0.001
Control	(Constant)	27.11	2.77		9.789	0.001
	The depression of hopelessness	−0.17	0.06	−0.23	−2.489	0.01
	Adaptation time	1.50	0.59	0.21	2.539	0.01
	Production age	0.004	0.06	0.005	0.059	0.95
	POSAS P	−0.05	0.08	−0.06	−0.664	0.50

^a^Predictors: (Constant), adaptation time with scars, age of scarring, hopelessness depression, POSAS patient. ^b^Dependent variable: psychosocial internalization of scars. ^c^Predictors: (Constant), age of scarring, time to adapt to scars, hopelessness depression, patient POSAS; F – ANOVA.

The coefficient of multiple determination (which represents the percentage of the dispersion of scar internalization explained by the joint action of the previously mentioned predictors) is *R*^2^ = 0.721 which indicates that the predictors contribute 72.1% to the dispersion of scar internalization. The percentage is highly significant for participants with posttraumatic scarring. The coefficient of multiple determination (*R*^2^) in the control (surgical) group is *R*^2^ = 0.324, which indicates that the predictors contribute 32.4% to the dispersion of scar internalization, so the possibility that other factors play a major predictive role.

The *t-*test of significance (excluding the intercept/constant) shows that the predictors contribute statistically significantly to the estimate of scar internalization in participants with posttraumatic scars.

Predictors for hopelessness depression (*β* = 0.19; *t* = 3.674; *p* < 0.001), scar adjustment time (*β* = 0.09; *t* = 2.217; *p* < 0.02), and participant appreciation of scars (*β* = 0.28; *t* = 5.067; *p* < 0.001) have the highest weight in the internalization of scars considering that they present the highest *β* value. In other words, predictors are relevant for scar integration but not for subsequent vulnerability from a psychopathological perspective. The predictor age of scar production (*β* = −0.13; *t* = −2.878; *p* < 0.005) has a negative relationship with the psychosocial internalization of scars, which points us toward a more efficient integration of scars with decreasing age (hypothetically they are easier to integrate in childhood).

Multivariate linear regression analysis was used to evaluate the independent predictor factors for the psychosocial internalization in the experimental and control groups.

Our results highlighted that in the experimental group, higher scores of depression, adaptation time and increased POSAS-P score increase the psychosocial internalization levels. Having the scar produced at older age decreases the internalization levels. Our regression equation proved to be a good fit for the model, explaining 51.6% of lack of hope and depression (*R*^2^ = 0.712).

In the control group, having more time to adapt to the scar increases the psychosocial internalization, while the higher depression lowers the internalization levels. The age at which the scar occurred and the POSAS level have a non-significant participation in psychosocial internalization. The results are presented in Table 7.

The t-significance test presented in Table 7 shows that some of the predictors contribute statistically significantly to the estimate of internalization of scars in participants with post-surgical scars (control group).

### 3.4. Predictors of prevalence by depression of hopelessness in participants with posttraumatic and postsurgical scars

We investigate predictors that we believe may play an important role in the development of hopelessness depression in participants with posttraumatic and surgical scars. The preliminary statistical analyses confirm compliance with the conditions regarding homogeneity and multicollinearity. Considering the associations between the variables presented during the research, we believe that the assessment of scars by the patient (POSAS patient), quality of life components such as: personal care (euroqol2), usual activities (euroqol3) and pain/discomfort (euroqol4) are important predictors in estimating cognitive depression (depression of hopelessness) in posttraumatic research participants.

In Table 8 we present the differences recorded through the statistical processing of the results depending on the presence or absence of depression [*F*(160) = 35.082; *p* < 0.001] in the experimental group and in the control group [*F*(130) = 18.632; *p* < 0.001].

**Table 8:** Coefficients of the factors included in the predictive multilinear regression equation in patients with scars (post-traumatic and surgical) according to hopelessness depression.

Model summary						
Group	*R*	*R* ^2^	Adjusted *R*^2^		*F*	
Experimental	0.729^a^	0.531	0.516		*F*_(160)_ = 35.082; *p* < 0.001	
Control	0.653^c^	0.427	0.404		*F*_(130)_ = 18.632; *p* < 0.001	
Group	Model	Unstandardized Coefficients		Standardized Coefficients	*t*	*p*
		*B*	Std. Error	Beta		
Experimental	(Constant)	−29.52	4.38		−6.726	0.001
	Personal care	9.82	4.61	0.15	2.130	0.03
	Usual activities	9.71	3.68	0.20	2.634	0.009
	Pain/discomfort	12.46	3.39	0.34	3.669	0.001
	POSAS patient	−0.30	0.09	−0.26	−3.056	0.003
Control	(Constant)	−25.85	5.95		−4.341	0.001
	Personal care	6.47	6.16	0.08	1.051	0.295
	Usual activities	9.32	5.62	0.18	1.656	0.100
	Pain/discomfort	6.91	4.89	0.17	1.415	0.160
	POSAS patient	0.04	0.10	0.04	0.473	0.637

^a^DV: hopelessness depression. ^b^(Constant), Predictors: (Constant), POSAS pacient, Personal care – EuroQoL 2, Usual activities – EuroQoL 3, Pain/discomfort – EuroQoL 4. ^c^Predictors: (Constant), POSAS patient, Personal care – EuroQoL 2, Usual activities – EuroQoL 3, Pain/discomfort – EuroQoL 4.

The coefficient of multiple determination (which represents the percentage of the dispersion of depression in people with scars) explained by the joint action of the predictors is *R*^2^ = 0.531 which indicates that the predictors contribute 53.1% to the dispersion of depression. The percentage recorded is highly significant for participants with post-traumatic scars (Table 8). The coefficient of multiple determination (*R*^2^) in the control (surgical) group is *R*^2^ = 0.427, which indicates that the predictors contribute 42.7% to the dispersion of depression, so the possibility exists that other factors play a major predictive role.

The t-test of significance presented in Table 8 (excluding the intercept/constant) shows that the predictors contribute statistically significantly to the estimate of depression in participants with posttraumatic scar and to a lesser extent in participants with surgical scar.

Predictors self-care ability (*β* = 0.15; *t* = 2.130; *p* < 0.03), usual activities (*β* = 0.20; *t* = 2.634; *p* < 0.009) and discomfort/pain (*β* = 0.34; *t* = 3.669; *p* < 0.001) have the highest weight in the internalization of scars considering that they present the highest β value. In other words, the predictors are relevant for depression of hopelessness and subsequent vulnerability from a psychopathological perspective. The predictor of participants’ appreciation of scars (*β* = −0.26; *t* = −4.341; *p* < 0.003) has a negative relationship with depression, which points us toward a more efficient integration of scars with a realistic appreciation of the consequences of trauma.

The values of the standardized coefficients (*β*) presented in Table 7 in the case of participants with posttraumatic scars show that a deterioration in the quality of life regarding personal care, usual activities and posttraumatic discomfort have a high potential to generate helplessness in people with scars in association with decreased realism regarding appreciation scars.

Multivariate linear regression analysis was used to evaluate the independent predictor factors for the development of depression (lack of hope) in our post-traumatic patients group.

Our results highlighted that in the experimental group, higher scores of self-care, usual activity, pain and discomfort increased the depression levels. Additionally, higher POSAS scores had a positive effect, as it proved to decrease the depression levels. Our regression equation proved to be a good fit for the model, explaining 51.6% of lack of hope depression (*R*^2^ = 0.516).

In the control group, none of these variables seemed to be independent risk factors for depression. The results are presented in Table 8.

The t significance test shows that the predictors do not contribute statistically significantly to the estimation of scar internalization in participants with post-surgical scar (control group).

The predictors of personal care, usual activities, pain/discomfort and patient’s assessment of scars are not relevant and are not predictive of the subsequent development of depression at least from a statistical perspective.

In the last part of the study we aimed to identify useful indicators and propose a practical working tool for doctors who estimate the impact of post-traumatic scars.

**Figure 1:**
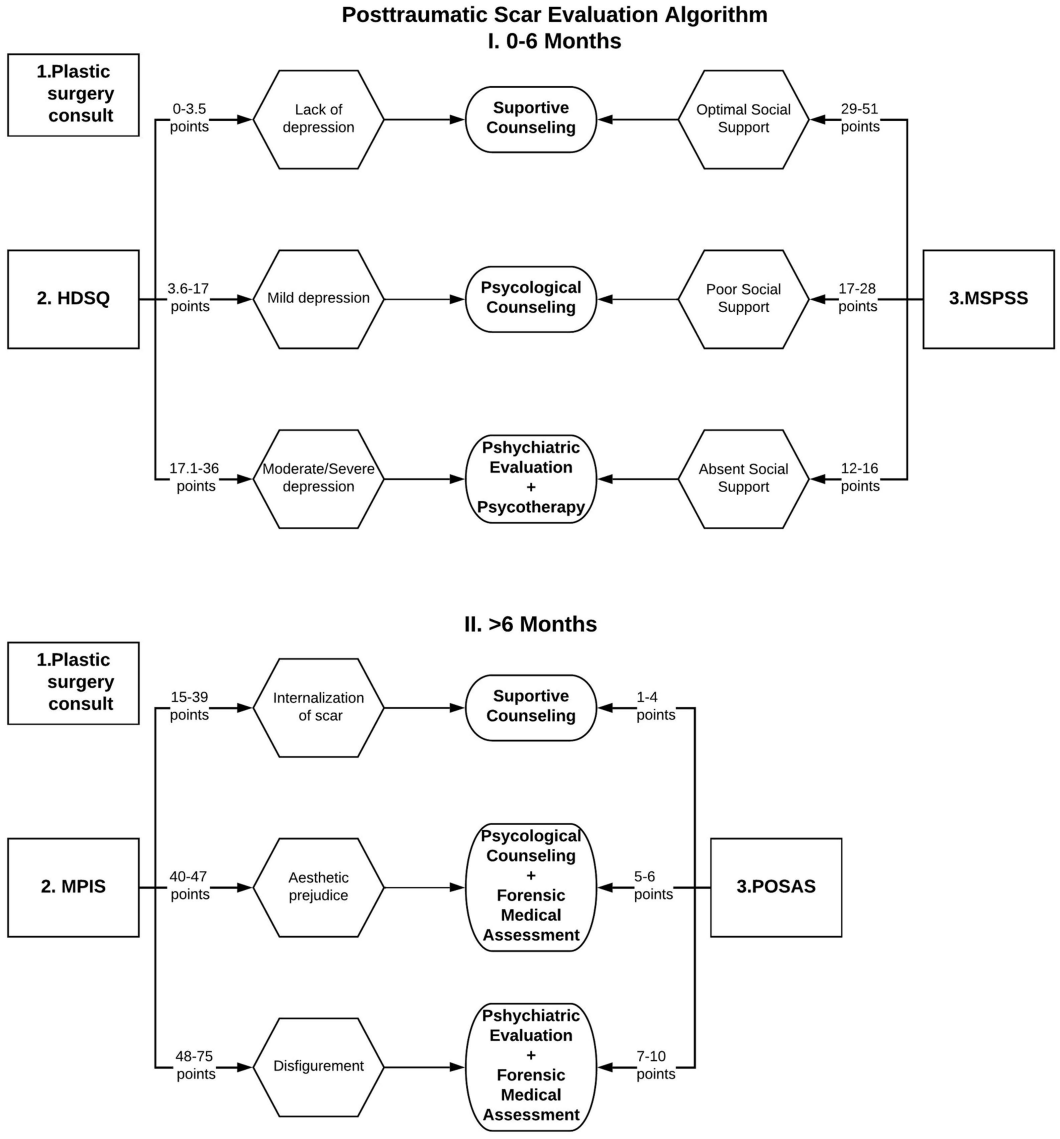
Posttraumatic scar evaluation algorithm.

To improve the management of patients with scars, we aimed to elaborate an algorithm, Figure 1, based on current and prior researches (1, 5), that could be used by medical examiner during the assessment of individuals with posttraumatic scars. Therefore, in case of scars that are more recent than 6 months (0–6), the evaluation should begin with a plastic surgery assessment, followed by the estimation of the severity of depressive symptoms and the quality of social support by employing the HDSQ, and MSPSS scales respectively, which are standards for quantifying the intensity of depression, namely absence of depression, moderate and severe depression, allowing adequate psychological and psychiatric decisions to be taken.

In the situation of older scars, lasting for more than 6 months, a plastic surgery re-assessment should be performed, followed by the quantifying of the scars internalization (MPIS) and patient’s appreciation of the scar (POSAS). Depending on patient’s results, a recommendation for psychological treatment (counseling or psychotherapy) or a psychiatric assessment, followed by specific therapy, and/or forensic medicine evaluation.

In Figure 1 are depicted the stages that should be followed by the forensic physician during the evaluation of an individual with post-traumatic scars.

## 4. Discussion

Our study aimed to highlight the differences between the impact of posttraumatic versus surgical scars on the psychological status of the patients.

The statistical analyses undertaken considered two groups (posttraumatic versus surgical) that are clinically, functionally, psychologically and socially differentiated. The presentation of the demographic aspects was the subject of laborious analyses presented in the first part of the research, where we used the statistical methods adapted to the congruent scientific requirements.

The visual appearance of scars varies greatly depending on the body location, individual characteristics and gender of the participant, the nature of the trauma and the healing conditions of the wounds causing itching, tension and pain. A significant part of the established and analyzed studies focused on the cellular mechanism (16–18) and clinimetric properties of scar rating scales like Vancouver Scar Scale, Visual Analog Scale, Seattle Scar Scale, POSAS (19–22), without taking into account psychopathological damages. We show that the association between the psycho-social internalization of scars and the depression of hopelessness (23) indicates a psychopathological orientation of people that (hopelessness) appears when the individual presents: a) negative expectations regarding the appearance of valued results to a high degree (expectation of a negative result) and b) negative expectations to change the probability of the occurrence of these results (24).

We demonstrate that some people experience psychological sequelae, including anxiety, depression, posttraumatic stress disorders, impaired self-esteem, and stigmatization. All these problems can have important effects on the patient’s quality of life as shown by (25), and on their health status potentially determining the occurrence of other health issues (31, 32). We argue that people with posttraumatic scarring stigmatize themselves to a greater extent than those with postsurgical scarring.

Van der Wal et al. (26, 32) and Chae et al. (27, 33) supported the arguments presented throughout the research showing similarly (but in a different line of research) that the post-traumatic patient’s opinion on the appearance of the scar (and not only the doctor’s) is an indicator of how the person will react in the future and of vulnerability to depressive mood or even depression.

The expectation of recovery, as well as social functionality, is much higher in the case of people who have gone through surgical interventions in comparison to people who have suffered unexpected traumas, so socio-professional tolerance is higher for those (13, 15).

Summarizing the presented results, we consider that, in the case of participants with post-traumatic scars (experimental group), they internalize the changes at the level of the skin depending on the appreciation of the scars measured with POSAS P (*β* = 0.28) and the lack of hope (*β* = 0.19). In other words, psychosocial internalization of scars is dependent on increased stigma, hopelessness, and adjustment time in the context of decreasing age at which the scars occurred (*β* = −0.13). In accordance with established definitions of the internalization of scars [2830] we consider that it could represent a habit that is dependent on the initial appearance of the person, the age and especially the gender of the person. Starting from the presented assumption, people with post-traumatic scars internalize scars to a greater extent, but with psychopathological implications.

The internalization of scars is dependent on the initial appearance of the person, the age and especially the gender of the person (6). Our results show that people with post-traumatic scars are oriented toward the internalization of scars, but also according to their shape and size. In other words, the patient’s attitude toward the appearance of the scar is an indicator of how the person will react in the future and of vulnerability. The obtained results support the previous statements in the sense that people with post-traumatic scars find their scars rather unpleasant.

In the situation where the scars were surgically produced (control group), the internalization of the scars is dependent on the time of adaptation to them (*β* = 0.21), i.e., with the passage of time the person will ignore the consequences of stigmatization. The decrease in hopelessness or the symptomatology of psychogenic depression (*β* = −0.23) will guarantee the integration of the scars into the body scheme and implicitly the internalization/acceptance of the scars.

A limited number of studies support the association between hopelessness depression and participant evaluation of scars (pain, itching, color, stiffness, thickness, surface, etc.) (29, 30). Concluding in consonance and in line with cognitive-social studies, a proximal and sufficient cause of (cognitive) depression symptoms is the expectation that desirable outcomes will not occur or aversive outcomes will occur for which no response from the personal history will change the probability of the occurrence of these outcomes (7, 14), eventually even resulting in associated organic damages (31, 32).

Cohen (15) believes that social support refers to “the provision of social support networks and psychological resources, deliberately, to benefit individuals with the skills necessary to adjust to stressful conditions.” Considering these aspects, we believe that social support is not perceived differently by people with surgical or post-traumatic scars precisely because of the availability and psychological resources of the group members (8, 14, 33).

We argue based on the registered coefficients that in people with scars there is an association with the tendencies toward isolation and loneliness at an emotional and social level, which will deeply affect the quality of life (34–36). Social loneliness is associated with the absence of employment in the social network and predominantly, with feelings of marginalization (13).

We believe that the availability of social support depends on the characteristics of people with scars as well as communication skills. Therefore, personal and social characteristics that make communication impossible are probably associated with psychopathological effects such as ambivalence in emotional expression, repressive defensiveness and fear of intimacy (13, 14, 37, 38).

Social support perceived by people with post-traumatic scars indicates a high perception (globally) that probably favors them (at the expense of surgical ones) due to the trauma, having the gratitude or support of others with a positive halo effect precisely through unpredictability (an accident) and involve a strong emotional impact. We consider this aspect to be positive at this stage of research given that social support often plays a buffering role (8, 14, 38).

Investigating the relationship between hopelessness depression and traumatic versus postsurgical scarring in the context of Abramson et al.’s theory (22) show that the maladaptive attributional style in a specific field (for example, the production of scars in a traumatic or post-surgical way) also entails vulnerability to depression when a person perceives social rejection as a result of the appearance of scars (39). Our results support previous assumptions in humans with posttraumatic scarring. Thus, motivational deficit, interpersonal dependence, psychomotor retardation, energy, apathy/anhedonia, insomnia, concentration difficulties and suicidal tendencies indicate a heightened vulnerability of people with post-traumatic scars that appeared contextually compared to people who present scars expected following surgical interventions.

Our results show that depression of hopelessness (as a subtype of depression) can be predicted by increased discomfort created by the scar, unfavorable appraisal of the scars, and decreased quality of life (36). In people with surgical scars, it is possible that different mechanisms intervene to stop the development of depression of hopelessness, possibly coping mechanisms in the current life situation (40, 41).

We postulate that patient assessment of scars, components of quality of life such as: personal care, usual activities and discomfort are important predictors in the estimation of cognitive depression (depression of hopelessness) in the case of posttraumatic scar research participants.

Posttraumatic Scar Evaluation Algorithm represents a new concept, for which we have designed a scar management protocol summarized in the diagram (Figure 1), used for the aesthetic prejudice evaluation. This algorithm proposes several steps that should be followed for the assessment of victims with post-traumatic injuries, depending on the age of the scars. It can also be considered a guide for the coroner in establishing aesthetic damage or disfigurement. The major advantage of this algorithm is the combination of physical assessment methods (POSAS) with psychosocial ones (MSPSS, HDSQ, MPIS).

Nowadays, in Romania, an old esthetic scale from 1973 is employed for the assessment of aesthetic damage (The aesthetic method derived from Greff’s and Hodin’s methods) (42). This scale includes only the assessment of the face, which it divides into 122 sectors plus correction coefficients and does not contain the interpretation of the obtained result (43). Another advantage of the algorithm proposed by us is the evaluation of scars located anywhere on the body.

## 5. Conclusion

People with scars show tendencies toward isolation and loneliness on an emotional and social level, which will deeply affect their quality of life. The visual appearance of scars varies greatly depending on the body location, individual characteristics and gender of the subject, the nature of the trauma and the healing conditions of the wounds causing itching, tension and pain. People with post-traumatic scars are oriented toward the internalization of scars, often depending on their shape and size. In other words, the patient’s attitude toward the appearance of the scar is an indicator of how the person will react in the future and of vulnerability. Hopeless depression (as a subtype of depression) can be predicted by increased discomfort created by the scar, unfavorable appraisal of the scars, and decreased quality of life. We proposed an algorithm for evaluating scars with the main goal of obtaining their early corrections but also with a view to decreasing internalization.

## Data availability statement

The datasets presented in this study can be found in online repositories. The names of the repository/repositories and accession number(s) can be found at: https://data.mendeley.com/datasets/7wx72rsjnk/1.

## Ethics statement

The studies involving human participants were reviewed and approved by the Ethics Committee of the County Emergency Clinical Hospital, Oradea, Nr. 23823/08.10.21. The patients/participants provided their written informed consent to participate in this study.

## Author contributions

GM, CB, CT, AC, MT, FM, CI, NP, IV, DD, and FV-M: conceptualization, validation, writing—original draft preparation, and visualization. GM, CT, and FV-M: methodology, resources, formal analysis, supervision, and project administration. GM and FV-M: software and investigation. GM, CT, MT, and FV-M: data curation, and writing—review and editing. University of Oradea: funding acquisition. All authors have read and agreed to the published version of the manuscript.

## Funding

This study was funded by the University of Oradea.

## Conflict of interest

The authors declare that the research was conducted in the absence of any commercial or financial relationships that could be construed as a potential conflict of interest.

## Publisher’s note

All claims expressed in this article are solely those of the authors and do not necessarily represent those of their affiliated organizations, or those of the publisher, the editors and the reviewers. Any product that may be evaluated in this article, or claim that may be made by its manufacturer, is not guaranteed or endorsed by the publisher.
